# Plasma ALKBH5 depletion during severe intermittent hypoxia and its attenuation by galectin-3 inhibition: an experimental rat study

**DOI:** 10.3389/fcvm.2026.1805064

**Published:** 2026-08-06

**Authors:** Saad Al-Anazi, Yasser A. Alshawakir, Syed Shahid Habib, Hayam Gad, Asma F. Alotaibi, Alanoud Aljasham, Wajd Althakfi, Mohamed A. Mekhtiche, Abeer Al-Masri

**Affiliations:** 1 Azeer Medical Company, Riyadh, Saudi Arabia; 2 Department of Physiology, College of Medicine, King Saud University, Riyadh, Saudi Arabia; 3 Histopathology Unit, Department of Pathology, College of Medicine, King Saud University, King Saud University Medical City, Riyadh, Saudi Arabia

**Keywords:** ALKBH5, experimental animal study, galectin-3, intermittent hypoxia, modified citrus pectin, obstructive sleep apnea, plasma biomarker

## Abstract

**Background:**

Intermittent hypoxia (IH), the pathophysiological hallmark of obstructive sleep apnea (OSA), promotes oxidative stress and cardiovascular remodeling. Galectin-3 (Gal-3) is a key mediator of hypoxia-associated inflammatory cardiac injury. ALKBH5, an m6A RNA demethylase detectable in plasma, has not been systematically characterized during hypoxic cardiovascular stress. This experimental study examined plasma ALKBH5 responses to graded IH and evaluated whether pharmacologic Gal-3 inhibition with modified citrus pectin (MCP) modulates circulating ALKBH5 levels and associated cardiac histopathology.

**Methods:**

Male Sprague–Dawley rats (*n* = 40) were randomized to five groups (*n* = 8/group): normoxia control; moderate IH (MIH; nadir FiO_2_ 10%–12%); severe IH (SIH; nadir FiO_2_ 5%–7%); MIH with MCP (800 mg/kg/day); and SIH with MCP. IH exposure was applied for 8 h/day over 10 days. Endpoint plasma ALKBH5 and Gal-3 concentrations were quantified by ELISA. Cardiac histology was assessed using blinded semi-quantitative injury scoring. Group comparisons were performed using Kruskal–Wallis tests with Dunn's *post-hoc* correction, and factorial effects were analyzed using aligned rank transform (ART) ANOVA. Correlations were explored using Spearman's rank correlation coefficients due to small sample size and non-normal distributions.

**Results:**

Plasma Gal-3 increased in a severity-dependent manner with IH (Control: 18.4 ± 3.2; MIH: 34.7 ± 5.8; SIH: 48.2 ± 7.1 ng/mL; *p* < 0.001) and was significantly reduced by MCP treatment (36%–45% reduction; *p* < 0.01), confirming effective target engagement. Plasma ALKBH5 [median (IQR)] was significantly reduced in SIH compared with controls [691 [562] vs. 2,048 [1,094] pg/mL; *p* = 0.008], while MIH showed a non-significant trend toward reduction [1,213 (951) pg/mL; *p* = 0.089]. MCP treatment was associated with higher plasma ALKBH5 concentrations in both IH groups [MIH + MCP: 1,866 [1,698]; SIH + MCP: 1,807 [1,595] pg/mL; *p* < 0.05 vs. respective IH groups]. ART ANOVA demonstrated a significant main effect of Gal-3 inhibition on circulating ALKBH5 independent of hypoxic severity [F(1,28) = 21.28, *p* < 0.001, partial *η*^2^ = 0.381]. Histological analysis revealed marked myocardial injury in untreated IH groups, whereas MCP-treated animals exhibited markedly attenuated histopathological injury within the limits of semi-quantitative histological assessment.

**Conclusion:**

Severe intermittent hypoxia has been linked to a drop in circulating ALKBH5 levels, whereas pharmacological inhibition of galectin-3 has been associated with maintaining plasma ALKBH5 concentrations and a significant reduction of hypoxia-induced myocardial pathological damage in an experimental rat model. These findings suggest circulating ALKBH5 as a hypoxia-responsive molecular signal controlled by inflammatory pathways; however, more mechanistic and longitudinal investigations are necessary to characterize its biological significance and possible translational value.

## 1 Introduction

Intermittent hypoxia (IH), the defining pathophysiological feature of obstructive sleep apnea (OSA), consists of recurrent cycles of oxygen desaturation and reoxygenation that activate oxidative stress pathways, neurohumoral responses, and pro-inflammatory signaling cascades (1). These processes are associated with negative cardiovascular outcomes, particularly arrhythmogenesis, myocardial remodeling, and progression of cardiac failure (2, 3). Despite the acknowledged association between obstructive sleep apnea (OSA) and cardiovascular morbidity (2, 4), there are currently few circulating biomarkers that accurately reflect the molecular consequences of IH-induced cardiac stress.

**Figure 1:**
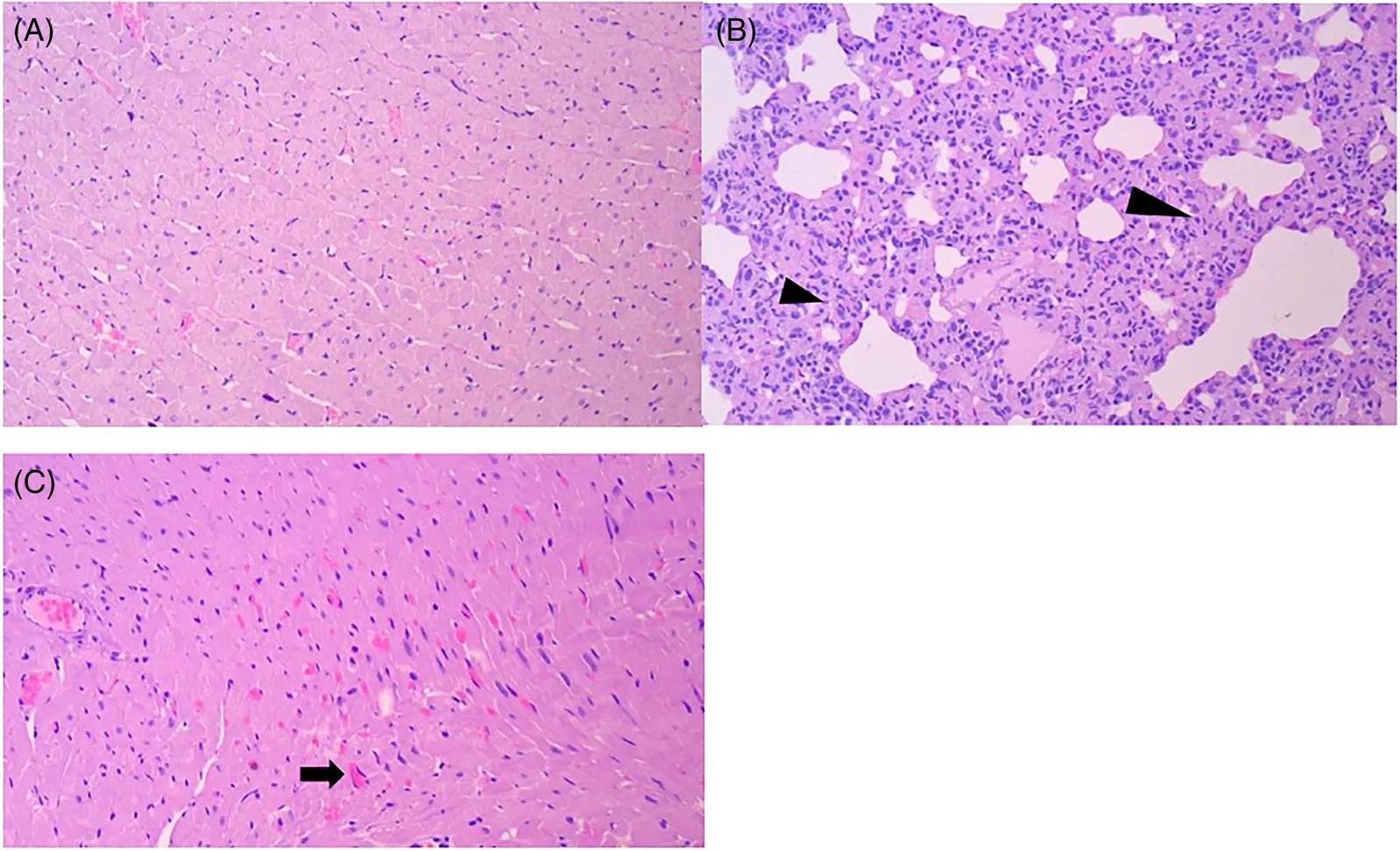
Representative light photomicrographs of H&E-stained cardiac sections. **(A)** Control hearts showed preserved myocardial architecture without notable inflammatory infiltration; **(B)** untreated SIH hearts showed interstitial edema, vascular congestion, and increased myofiber separation; **(C)** SIH + MCP hearts showed markedly attenuated injury with reduced edema and better-preserved architecture compared with untreated SIH. These findings are qualitative/descriptive in the current dataset.

Galectin-3 (Gal-3), a β-galactoside-binding lectin, is recognized for its role in fibrogenesis and inflammation. In clinical populations, greater levels of circulating Gal-3 are linked to more severe OSA and a higher risk of cardiac disease (5, 6). Experimental studies indicate that Gal-3 has a role in macrophage activation, myofibroblast proliferation, and the accumulation of extracellular matrix inside the myocardium, hence promoting structural remodeling in response to pathological stress conditions (7, 8).Pharmacologic inhibition of Gal-3 by utilizing modified citrus pectin (MCP), a competitor inhibitor of its carbohydrate recognition domain, has been shown to attenuate cardiac remodeling in models of pressure overload, and inflammatory injury (7–9).

It is increasingly recognized that epitranscriptomic regulation, namely N6-methyladenosine (m6A) RNA modification, serves as a crucial modulator of cellular stress adaptation. ALKBH5 is an α-ketoglutarate-dependent dioxygenase that demethylates m6A. It changes the stability and efficiency of mRNA translation in response to reduced oxygen levels and inflammation (10–12). ALKBH5 mostly resides in the nucleus; nevertheless, it has been identified in extracellular vesicles during inflammatory and stress-related conditions (13). Intracellular ALKBH5 has been shown to stabilize hypoxia-responsive transcripts in oncogenic and proliferative contexts (10, 11, 14); moreover, the regulation potential significance of circulating ALKBH5 under cardiovascular stress remain unclear.

Recent findings suggest potential relationships between Gal-3–mediated inflammatory signaling and cellular stress responses (1, 15), indicating that the manipulation of Gal-3 activity may affect systemic molecular pathways. The impact of Gal-3 inhibition on circulating ALKBH5 levels under hypoxic cardiovascular stress has not been previously investigated.

Accordingly, we hypothesized that: (1) exposure to severe intermittent hypoxia is associated with reduced circulating ALKBH5 concentrations in a severity-dependent manner; and (2) pharmacologic inhibition of Gal-3 with modified citrus pectin modulates plasma ALKBH5 levels and attenuates hypoxia-associated cardiac histopathological changes in rats exposed to graded IH.

## 2 Materials and methods

The overall experimental design and study workflow are illustrated in Figure 1.

### 2.1 Study design and ethical approval

This controlled experimental study with adult male Sprague–Dawley rats (300–400 g; King Saud university Laboratories) were housed under standard conditions (12 h light/dark cycle, 22 ± 2 °C, 50 ± 10% humidity) with *ad libitum* access to standard chow and water. All procedures were approved by the Institutional Animal Care and Use Committee (Protocol No. KSU-SE-24-21) and conducted in accordance with the Guide for the Care and Use of Laboratory Animals (National Research Council, (32)) and ARRIVE 2.0 guidelines (16).

### 2.2 Randomization and group allocation

Animals were randomized via computer-generated block randomization (block size=5, stratified by body weight) into five experimental groups (*n* = 8/group):

1. Control (CON): Continuous normoxia (21% FiO_2_)
2. Moderate IH (MIH): Nadir FiO_2_ 10%–12%, 15–30 cycles/hour
3. Severe IH (SIH): Nadir FiO_2_ 5%–7%, 30–60 cycles/hour
4. MIH + MCP: Moderate IH with Gal-3 inhibition
5. SIH + MCP: Severe IH with Gal-3 inhibition

Investigators performing ELISA assays, histological scoring, and statistical analyses were blinded to group allocation until completion of primary analyses.

### 2.3 Intermittent hypoxia protocol

IH was delivered using a custom-built control chamber developed in our laboratory over 10 consecutive days (08:00–16:00 daily, 8 h/day). Each hypoxic cycle consisted of a linear decrease from 21% to target nadir FiO₂ over 60–90 s, maintained for 30 s, followed by reoxygenation to 21% over 30–60 s. Control animals were housed in identical chambers with continuous room air circulation.

### 2.4 Galectin-3 inhibition and pharmacodynamic validation

Modified citrus pectin (PectaSol-C, EcoNugenics; molecular weight 10–30 kDa, degree of esterification <10%) was dissolved fresh daily in sterile 0.9% saline and administered via intraperitoneal injection (800 mg/kg) 30 min prior to daily IH exposure (17). Dose selection was based on previous *in vivo* studies demonstrating that systemic MCP at 200–800 mg/kg attenuates organ injury through Gal-3 inhibition. Control and untreated IH groups received equivalent volumes of sterile saline (9).

#### 2.4.1 Target engagement assessment

To verify pharmacologic inhibition, plasma Gal-3 concentrations were measured by rat-specific ELISA (R&D Systems, Quantikine DY2484) at endpoint. Successful inhibition was predefined as ≥30% reduction in circulating Gal-3 versus untreated IH groups.

### 2.5 Endpoint sample collection

At day 10 (16–18 h following the final IH exposure to avoid acute stress artifacts), rats were anesthetized with isoflurane (3% in 100% O₂). Blood (3 mL) was collected via cardiac puncture into pre-chilled K₂EDTA tubes. Plasma was isolated by centrifugation (3,000  ×  g, 15 min, 4 °C), aliquoted, and stored at −80 °C until analysis. Samples underwent a single freeze-thaw cycle. Hearts were immediately exercised and processed for histology (18).

### 2.6 Biomarker quantification

Plasma ALKBH5 was measured in duplicate using a commercially available rat-specific ELISA [MyBioSource, MBS2615604; analytical range 1.56–100 ng/mL; intra-assay coefficient of variation (CV) < 5%, inter-assay CV <8%]. Plasma Gal-3 was quantified using the Quantikine rat Galectin-3 immunoassay (intra-assay CV <4.5%, inter-assay CV <7%). Optical density was determined at 450 nm with wavelength correction at 540 nm using a microplate spectrophotometer. Concentrations were calculated from four-parameter logistic standard curves.

### 2.7 Cardiac histology and quantitative injury scoring

Cardiac histology was evaluated on transverse ventricular sections. Cardiomyocyte cross-sectional area was measured as an indicator of hypertrophy by tracing at least 50 myocytes per section from the mid-myocardial left ventricle using ImageJ software (NIH, USA). Inflammatory cell infiltration and interstitial edema were graded semi-quantitatively following previously published criteria (Table 1) adapted from Castro-Grattoni et al. (19).

**Table 1:** Cardiac injury scoring criteria.

Score	Myocardial Alterations
0 = Absent	Normal fibers, no edema or inflammatory cells
1 = Mild	Focal interstitial edema or scattered mononuclear cells
2 = Moderate	Multifocal edema with small clusters of inflammatory cells
3 = Severe	Diffuse edema, vascular congestion, and dense inflammatory infiltration with myocyte degeneration

### 2.8 Statistical analysis

Statistical analyses were performed using R software (version 4.3.1). Data normality was assessed with the Shapiro–Wilk test and homogeneity of variance with Levene's test. Because ALKBH5 exhibited non-normal distributions and non-homogeneous variances across groups, comparisons were conducted using Kruskal–Wallis H-tests followed by Dunn's *post hoc* pairwise comparisons with Benjamini–Hochberg adjustment for multiple testing.

Factorial effects of intermittent hypoxia (IH) severity and Gal-3 inhibition among IH-exposed groups were evaluated using Aligned Rank Transform (ART) ANOVA, as described by Jacob (31). Histology scores, which met assumptions of normality and homogeneity of variance, were analyzed using one-way ANOVA followed by Tukey's *post hoc* test. A two-sided *p* value < 0.05 was considered statistically significant.

Correlations between plasma ALKBH5 concentrations and physiological parameters (heart rate, blood pressure, oxygen saturation, respiratory rate, and body weight) were evaluated within the severe intermittent hypoxia (IH) subgroups (SIH and SIH + MCP) using Spearman's rank correlation coefficients (*ρ*). Spearman's method was chosen due to the small sample size (*n* = 8 per group) and non-normal variable distributions. Given the exploratory and descriptive nature of these analyses, *p*-values were not adjusted for multiple comparisons; however, exact *p*-values are reported. A two-tailed significance level of *p* < 0.05 was adopted for all tests.

A *post hoc* power analysis (G*Power 3.1.9.7) for the primary factorial comparison [Aligned Rank Transform (ART) ANOVA] indicated >95% power to detect the observed effect size (partial *η*^2^ = 0.381) with α = 0.05 and *n* = 8 per group.

## 3 Results

### 3.1 Pharmacodynamic validation of galectin-3 inhibition

Plasma galectin-3 (Gal-3) concentrations differed significantly across experimental groups (Table 2). Gal-3 levels increased in a severity-dependent manner with intermittent hypoxia (IH), with severe IH producing the highest concentrations (48.2 ± 7.1 ng/mL), representing an approximately 2.6-fold increase compared with normoxic controls (18.4 ± 3.2 ng/mL; *p* < 0.001). Moderate IH was also associated with elevated Gal-3 levels relative to controls (34.7 ± 5.8 ng/mL; *p* < 0.001).

**Table 2:** Plasma galectin-3 concentrations (ng/mL).

Group	Mean ± SD	Median (IQR)	*p*-value vs. Control	*p*-value vs. IH counterpart
Control	18.4 ± 3.2	17.8 (4.2)	—	—
MIH	34.7 ± 5.8*	33.2 (7.1)	<0.001	—
SIH	48.2 ± 7.1*	47.5 (8.3)	<0.001	—
MIH + MCP	22.1 ± 4.1^†^	21.4 (5.2)	0.089	0.002
SIH + MCP	26.3 ± 5.4^†‡^	25.1 (6.8)	0.008	<0.001

*p < 0.001 vs. Control.

† *p* < 0.01 vs. respective IH group.

‡ *p* < 0.05 vs. Control.

Treatment with modified citrus pectin (MCP) was associated with significantly lower circulating Gal-3 concentrations in both IH groups. Gal-3 levels were reduced by 36% in the MIH + MCP group (22.1 ± 4.1 ng/mL; *p* = 0.002 vs. MIH) and by 45% in the SIH + MCP group (26.3 ± 5.4 ng/mL; *p* < 0.001 vs. SIH), indicating effective pharmacodynamic modulation of circulating Gal-3.

### 3.2 Plasma ASLKBH5 concentrations

Endpoint plasma ALKBH5 concentrations differed significantly among experimental groups (Kruskal–Wallis *χ*^2^ = 16.695, df = 4, *p* = 0.002; Table 3). Severe IH was associated with a marked reduction in circulating ALKBH5 compared with normoxic controls [median [IQR]: 691 [562] vs. 2,048 [1,094] pg/mL; *p* = 0.008]. Moderate IH showed a numerical decrease in ALKBH5 concentrations; however, this difference did not reach statistical significance after correction for multiple comparisons [1,213 (951) pg/mL; *p* = 0.089 vs. control].

**Table 3:** Plasma ALKBH5 concentrations at Day 10.

Group	*n*	Mean ± SD (pg/mL)	Median (IQR) (pg/mL)
Control	8	2,851 ± 3,126	2,047.75 (1,094.09)
MIH	8	1,137 ± 536	1,213.31 (950.56)
SIH	8	699 ± 393*	691.15 (561.94)
MIH + MCP	8	2,477 ± 1,748^†^	1,865.66 (1,698.48)
SIH + MCP	8	2,017 ± 985^†^	1,806.82 (1,594.67)

*p = 0.008 vs. Control.

† *p* < 0.05 vs. respective IH group (Dunn's test with Benjamini-Hochberg correction).

MCP treatment was associated with higher plasma ALKBH5 concentrations in both IH groups. In the severe IH condition, MCP increased median ALKBH5 levels to 1,807 pg/mL (*p* = 0.015 vs. untreated SIH), with values not significantly different from controls (*p* = 0.42). A similar increase was observed in the moderate IH group treated with MCP (1,866 pg/mL; *p* = 0.012 vs. untreated MIH).

### 3.3 Factorial analysis of treatment effects

Among IH-exposed animals (*n* = 32), aligned rank transform (ART) ANOVA demonstrated a significant main effect of Gal-3 inhibition on circulating ALKBH5 concentrations [F(1,28) = 21.28, *p* < 0.001, partial *η*^2^ = 0.381; Table 4]. Neither the main effect of IH severity [F(1,28) = 1.46, *p* = 0.236] nor the interaction between IH severity and Gal-3 inhibition [F(1,28) = 0.20, *p* = 0.662] reached statistical significance.

**Table 4:** Two-way ART ANOVA results for IH-exposed groups.

Source	df	F	*p*-value	Partial *η*^2^
Gal-3 Inhibition	1.28	21.28	<0.001	0.381
IH Severity	1.28	1.46	0.236	0.050
IH × Gal-3 Interaction	1.28	0.20	0.662	0.007

### 3.4 Cardiac histopathology

Semi-quantitative histological assessment demonstrated increased myocardial structural abnormalities in IH-exposed animals compared with controls (Table 5). Untreated severe IH was associated with the highest composite injury scores (7.17 ± 0.75), which were significantly greater than those observed in moderate IH (4.67 ± 2.94; *p* = 0.03) and normoxic controls (0.25 ± 0.16; *p* < 0.001). Histopathological features included interstitial edema, vascular congestion, inflammatory infiltration, and myofiber separation.

**Table 5:** Semi-quantitative cardiac injury scores.

Group	Edema	Congestion	Inflammation	Myofiber Separation	Composite Score
Control	0.1 ± 0.2	0.1 ± 0.1	0.0 ± 0.0	0.1 ± 0.2	0.3 ± 0.2
MIH	1.8 ± 0.8	1.4 ± 0.5	0.6 ± 0.5	0.9 ± 0.6	4.7 ± 2.9
SIH	2.4 ± 0.5	2.1 ± 0.4	1.4 ± 0.5	1.3 ± 0.5	7.2 ± 0.8
MIH + MCP	0.2 ± 0.2	0.2 ± 0.2	0.2 ± 0.2	0.2 ± 0.2	0.8 ± 0.4^†^
SIH + MCP	0.3 ± 0.2	0.2 ± 0.3	0.3 ± 0.3	0.2 ± 0.2	1.0 ± 0.5^†^

† *p* < 0.001 vs. respective IH group (ANOVA with Tukey's post-hoc); scores represent mean ± SD. Values of 0.8–1.0 indicate marked attenuation rather than complete elimination of injury.

In contrast, MCP-treated IH groups exhibited **markedly attenuated histopathological injury** compared with their respective untreated IH counterparts (*p* < 0.001), with composite scores approaching control levels **within the limits of semi-quantitative histological assessment** (0.8–1.0 vs. 0.3 ± 0.2; *p* > 0.05 vs. controls).

### 3.5 Correlations between plasma ALKBH5 and physiological parameters

Exploratory descriptive correlation analyses were performed within the severe IH subgroups (Table 6). In untreated SIH animals, plasma ALKBH5 concentrations showed no significant correlations with measured physiological parameters (all |*ρ*| < 0.30, *p* > 0.40). In contrast, in the SIH + MCP group, plasma ALKBH5 demonstrated a positive correlation with heart rate (*ρ* = 0.714, *p* = 0.047) and a trend-level positive association with peripheral oxygen saturation (SpO₂; *ρ* = 0.671, *p* = 0.069). No significant correlations were observed for body weight, blood pressure, or respiratory rate in either subgroup.

**Table 6:** Spearman's rank correlation coefficients (*ρ*) between plasma ALKBH5 and physiological parameters in severe IH groups.

Physiological Parameter	SIH (Untreated)	SIH + MCP (Treated)
	*ρ* (*p*-value)	*ρ* (*p*-value)
Body Weight	0.180 (0.670)	−0.238 (0.570)
Systolic Blood Pressure	0.120 (0.780)	0.476 (0.233)
Diastolic Blood Pressure	0.084 (0.844)	0.381 (0.352)
Heart Rate	−0.071 (0.867)	0.714 (0.047)*
SpO₂	−0.061 (0.885)	0.671 (0.069)^†^
Respiratory Rate	−0.071 (0.867)	0.240 (0.568)

Correlations are exploratory and descriptive, not adjusted for multiple testing.

*Significant correlation at *p* < 0.05.

† Trend-level correlation (*p* < 0.10).

## 4 Discussion

In this controlled experimental animal study, we **show** that severe intermittent hypoxia (IH) is associated with a significant reduction in circulating ALKBH5, an m6A RNA demethylase, and that pharmacological inhibition of galectin-3 (Gal-3) using modified citrus pectin (MCP) attenuates this reduction while substantially limiting IH-associated myocardial histopathological injury. These findings **support** a link between hypoxia-driven inflammatory signaling and systemic epitranscriptomic alterations, while remaining exploratory with respect to underlying mechanisms.

### 4.1 Severity-dependent effects of intermittent hypoxia on circulating ALKBH5

A key finding of this study is that ALKBH5 depletion mostly occurred under severe IH circumstances, while moderate IH resulting in a barely minor numerical reduction. This pattern indicates that circulating ALKBH5 may be responsive to the severity of hypoxic burden, aligning with previous research that illustrates threshold-dependent activation of oxidative stress, sympathetic drive, and inflammatory cascades in models of intermittent hypoxia (IH) and obstructive sleep apnea (OSA) (20, 21).

Previous investigations of ALKBH5 have predominantly focused on intracellular regulation within cancer biology, wherein hypoxia stimulates ALKBH5 expression to facilitate transcriptome adaptability and cellular survival (22). Conversely, the current findings relate to circulating ALKBH5, which presumably indicates different biological processes. Although conjectural, intense systemic hypoxic stress may modify protein release, stability, or clearance dynamics, resulting in diminished measurable plasma levels. The present study does not demonstrate causation or functional implications of ALKBH5 depletion; the observed alterations should be regarded as associative rather than mechanistically conclusive. Whether circulating ALKBH5 reflects active biological signaling or passive release secondary to cellular stress remains unknown.

### 4.2 Effect of galectin-3 inhibition on ALKBH5 under intermittent hypoxia

Gal-3 inhibition using MCP resulted in a significant attenuation of IH-associated ALKBH5 reduction across both moderate and severe IH exposures. This effect was observed in parallel with a confirmed reduction in circulating Gal-3, consistent with prior experimental studies demonstrating effective Gal-3 pathway modulation using MCP (23, 24). Gal-3 is a recognized mediator of macrophage activation, extracellular matrix remodeling, and inflammatory amplification in cardiovascular disease (25). While the present study was not designed to define molecular mechanisms, it is **plausible** that attenuation of inflammatory burden may secondarily influence the stability or detectability of circulating ALKBH5. Potential mechanisms—including reduced proteolytic degradation, altered extracellular vesicle trafficking, or indirect effects of reduced oxidative stress—remain hypothesis-generating and not directly tested, requiring direct experimental validation (26).

### 4.3 Cardiac histopathology in intermittent hypoxia and Gal-3 inhibition

In accordance with previous IH models, untreated IH exposure resulted in myocardial edema, vascular congestion, inflammatory infiltration, and myofiber separation, with the severity of damage according to hypoxic load (27). Animals administered MCP had significantly reduced histopathological adverse effects relative to untreated IH groups, suggesting considerable structural preservation within the confines of semi-quantitative histological evaluation.

These results support previous studies indicating that Gal-3 inhibition alleviates myocardium remodeling in models of pressure overload and metabolic stress (28). Due to the semi-quantitative nature of histologic scoring and the lack of functional cardiac measurements, the protective effect observed should be regarded as a reduction of injury rather than conclusive cardioprotection or total avoidance of disease.

### 4.4 Relationship between ALKBH5 and physiological parameters

Exploratory descriptive correlation analyses revealed that associations between circulating ALKBH5 and selected physiological parameters emerged only in MCP-treated severe IH animals. No such relationships were observed in untreated severe IH. Similar dissociation between biomarkers and physiological control has been reported in advanced hypoxia-related cardiovascular dysfunction, where inflammatory signaling disrupts normal regulatory coupling (30).

While these findings suggest that modulation of inflammatory signaling may influence systemic biomarker–physiology relationships, the small sample size and exploratory design preclude firm conclusions. These observations should therefore be considered descriptive and hypothesis-generating, warranting confirmation in larger, independently powered studies.

### 4.5 Study limitations

It is important to recognize several limitations. The work utilized a subacute IH exposure spanning 10 days, in contrast to OSA, which is a chronic illness; hence, extended models are necessary to evaluate persistent ALKBH5 dynamics and structural remodeling. Second, downstream inflammatory mediators and myocardial m6A alterations were not quantified, constraining mechanistic interpretation. Third, ELISA was used to measure circulating ALKBH5. This test fails to identify the difference between free protein and extracellular vesicle-associated forms, which may have distinct biological significance (29). Finally, correlation analyses were exploratory and not corrected for multiple testing.

### 4.6 Implications and future directions

These findings suggest that circulating ALKBH5 may represent a hypoxia-responsive molecular signal that is altered by severe IH and modulated by Gal-3 inhibition. While the biological role of extracellular ALKBH5 remains undefined, these results provide a rationale for future studies examining its regulation, tissue origin, and potential relationship to cardiovascular stress responses. Translation to clinical OSA populations will require rigorous validation, longitudinal assessment, and evaluation of predictive value before any biomarker application can be considered. Future required investigations, including:

- Quantification of ALKBH5 and Galectin-3 mRNA and protein expression in lung tissue and in both left and right ventricular myocardium,
- Correlation analyses between tissue expression and circulating ALKBH5 levels,
- Inclusion of additional organs (e.g., liver, kidney, and circulating immune cells) to identify potential systemic contributors, and
- Cellular localization studies to define the specific cell types responsible for ALKBH5 expression and release under intermittent hypoxic stress.

## 5 Conclusion

Severe intermittent hypoxia was associated with a significant reduction in circulating ALKBH5, whereas pharmacologic inhibition of galectin-3 was associated with attenuation of ALKBH5 depletion and marked reduction of myocardial histopathological injury. The emergence of associations between ALKBH5 and selected physiological parameters in treated animals suggests that inflammatory modulation may influence systemic biomarker–physiology relationships under hypoxic stress. Collectively, these findings support further investigation into the regulation, tissue origin, and relevance of circulating ALKBH5 in chronic hypoxia-related cardiovascular disease.

## Data Availability

The raw data supporting the conclusions of this article will be made available by the authors, without undue reservation.
