# Correction: Plasma ALKBH5 depletion during severe intermittent hypoxia and its attenuation by galectin-3 inhibition: an experimental rat study

**DOI:** 10.3389/fcvm.2026.1997459

**Published:** 2026-10-06

**Authors:** Saad Al-Anazi, Yasser A. Alshawakir, Syed Shahid Habib, Hayam Gad, Asma F. Alotaibi, Alanoud Aljasham, Wajd Althakfi, Mohamed A. Mekhtiche, Abeer Al-Masri

**Affiliations:** 1 Azeer Medical Company, Riyadh, Saudi Arabia; 2 Department of Physiology, College of Medicine, King Saud University, Riyadh, Saudi Arabia; 3 Histopathology Unit, Department of Pathology, College of Medicine, King Saud University, King Saud University Medical City, Riyadh, Saudi Arabia

**Keywords:** ALKBH5, experimental animal study, galectin-3, intermittent hypoxia, modified citrus pectin, obstructive sleep apnea, plasma biomarker

Affiliation Department of Physiology, College of Medicine, King Saud University, Riyadh, Saudi Arabia was erroneously given as Department of Physiology, College of Medicine, King Saud University, Saudi Arabia Azeer Medical Company, Riyadh, Saudi Arabia.

Affiliation Histopathology Unit, Department of Pathology, College of Medicine, King Saud University, King Saud University Medical City, Riyadh, Saudi Arabia was omitted from the paper. This affiliation has now been added for author Wajd Althakfi.

Affiliation Azeer Medical Company, Riyadh, Saudi Arabia was omitted from the paper. This affiliation has now been added for author Saad Al-Anazi. The conflict of interest statement has been corrected to “Author SA-A was employed by Azeer Medical Company. The remaining author(s) declared that this work was conducted in the absence of any commercial or financial relationships that could be construed as a potential conflict of interest.”

