# Supplementary material for: Plasma ALKBH5 depletion during severe intermittent hypoxia and its attenuation by galectin-3 inhibition: an experimental rat study
Source: Front Cardiovasc Med. 2026 Aug 6;13:1805064. doi: 10.3389/fcvm.2026.1805064 (PMC13490193; doi:10.3389/fcvm.2026.1805064)
Supplement: Supplementary file 1 [file Image1.pdf]

**Supplementary Material**

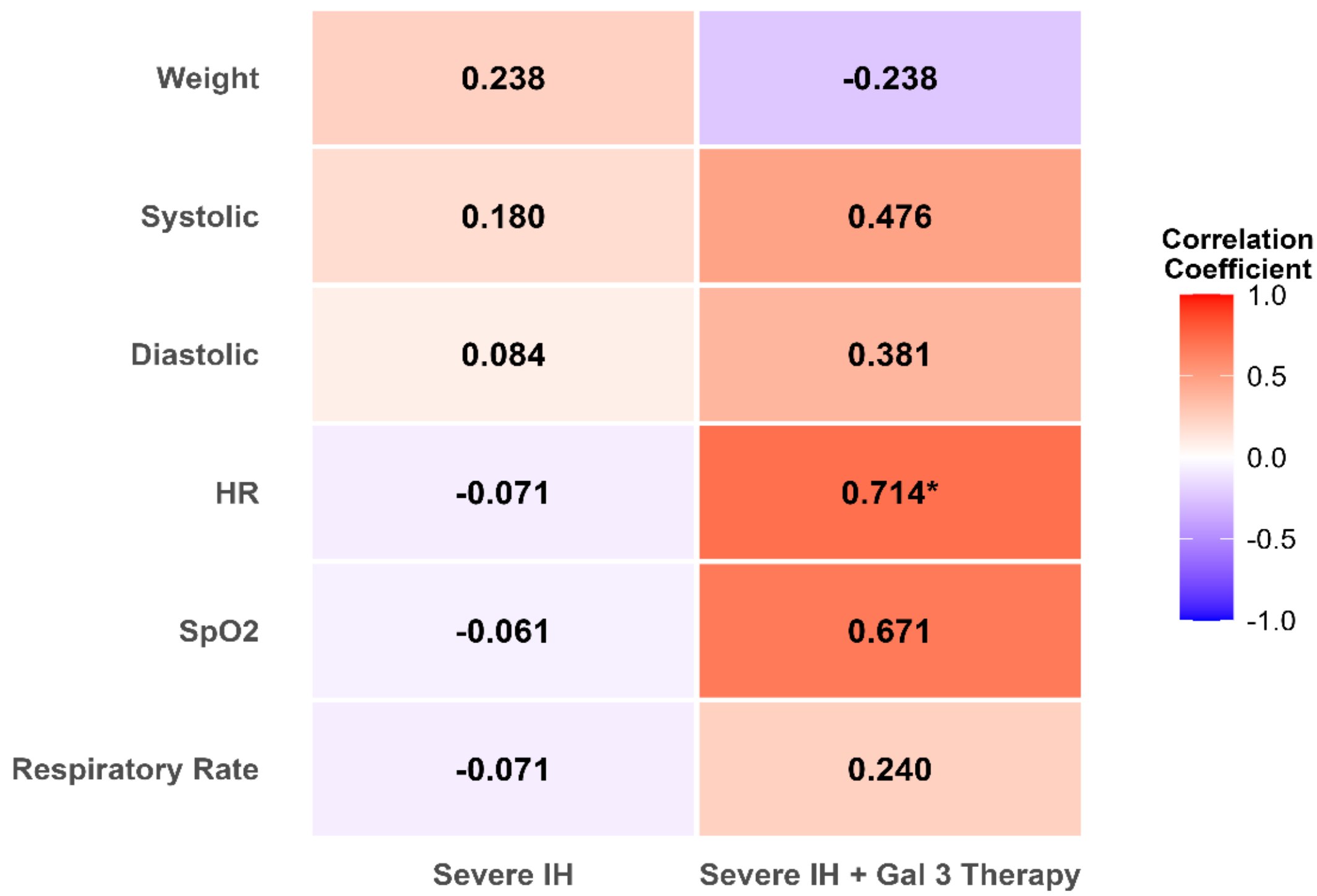


**Supplementary Figure 1. Correlation heatmap of physiological parameters and ALKBH5 levels in severe IH groups.** Color intensity indicates strength of Spearman's rank correlation (ρ). In SIH+MCP animals, ALKBH5 shows a positive correlation with heart rate (ρ=0.714) and oxygen saturation ($SpO_2$, ρ=0.671). Correlations are exploratory and descriptive, not adjusted for multiple testing.
